# Is concentration an indirect link between social anxiety and educational achievement in adolescents?

**DOI:** 10.1371/journal.pone.0249952

**Published:** 2021-05-14

**Authors:** Eleanor Leigh, Kenny Chiu, David M. Clark

**Affiliations:** 1 Department of Experimental Psychology, University of Oxford, Oxford, United Kingdom; 2 Department of Psychology, Institute of Psychiatry, Psychology & Neuroscience, King’s College London, London, United Kingdom; Australian National University, AUSTRALIA

## Abstract

Social anxiety is associated with reduced educational achievement. Given that concentration is a predictor of educational achievement, and social anxiety symptoms are associated with reduced concentration in class, this prospective study examined the possibility that social anxiety may impair educational achievement through reduced classroom concentration. A sample of 509 participants (53.8% female; *M* age: 12.77 years [*SD* = 0.81]) recruited from secondary schools completed questionnaires assessing social anxiety symptoms, depressive symptoms, and concentration in class. Educational achievement was assessed by internal grades within schools. An indirect effect of social anxiety on later educational achievement via concentration was observed, over and above baseline achievement and depression symptoms; adolescents with higher levels of social anxiety tend to have more difficulties concentrating in class, which in turn is associated with poorer academic outcomes. Findings underscore the challenges socially anxious adolescents will face trying to learn in school, and the need for education providers and clinicians to consider the effect of social anxiety symptoms on concentration and learning.

## Introduction

Educational achievement is an important determinant of life outcomes, including future occupational status [1], physical health, and even life expectancy [2]. A number of factors have been established as important in predicting educational achievement, ranging from fluid intelligence [3], to personality traits [4] and systemic factors [5, 6]. One further area of research that has received relatively little attention is the possible impact of social anxiety symptoms on educational achievement [7]. Such symptoms are common and typically first occur in early adolescence [8], and although some studies have observed that social anxiety symptoms are associated with reduced educational achievement (e.g. [9]), the mechanisms by which they interfere with young peoples’ learning abilities are unclear. It has been suggested that concentration may be one such mechanism [10]: concentration is a predictor of educational achievement [11] and social anxiety symptoms are associated with reduced concentration [12]. However, this hypothesized pathway has not yet been tested empirically, and as such, the current study aimed to examine whether social anxiety symptoms are associated with poorer educational achievement via reduced concentration in the classroom.

### Social anxiety and educational achievement

Several cross-sectional studies have found that a diagnosis of social anxiety disorder (SAD) is associated with indices of reduced educational achievement, including lower self-reported grades [13], lower objective examination results [9, 14], and difficulty completing assignments [15]. On the other hand, other studies have failed to find a direct association between social anxiety and educational achievement. For example, Strahan [16] reported a non-significant prospective association between baseline social anxiety and grade point average at two-year follow-up in a sample of college students. Likewise, a prospective study of Finnish adolescents reported a non-significant association between a diagnosis of SAD and academic grade [17]. Finally, in a cross-sectional study of 805 high school students, Scanlon, Del Toro [18] did not find evidence for a direct association between social anxiety and science grade. It would appear that although there is some evidence for a direct association, this has not always been replicated.

Increasingly, interest has turned to the possible indirect associations between anxiety and educational achievement. Previous reviews examining the associations between anxiety and attainment have proposed the existence of multiple indirect pathways [7, 10], including school absence and homework avoidance. Similar pathways may be present in the association between social anxiety and educational achievement. In the study of Scanlon, Del Toro [18] with unselected adolescents, whilst no direct association was found, the authors did observe a significant indirect association between social anxiety symptoms and science grade via reduced social engagement. In addition, they found that social anxiety symptoms were associated with lower peer support, which led to lower social engagement and then lower science grade. It may be that more socially anxious individuals hold back from engaging in classroom learning activities and peer-learning opportunities, which in turn impacts on their educational achievement. One possible indirect pathway that has been proposed but remains untested is concentration problems [10].

### Social anxiety symptoms and concentration

Concentration is an attentional process that involves the ability to focus on a particular task [19]. Concentration problems are commonly reported in anxiety disorders, for example, nearly half of parents of anxiety disordered youth report this as an issue [15]. Furthermore, experimental studies have demonstrated the detrimental effect of anxiety on concentration and attention [20, 21]. Concentration difficulties may be particularly relevant to social anxiety. Classrooms are fundamentally social and performance settings; young people are surrounded by their peers and may well be expected to answer a question, read aloud or write information on the whiteboard. Situations such as these are likely to generate anxiety for socially anxious individuals [22]. A number of studies have shown that socially anxious individuals turn their attention inwards in difficult social situations, focusing more on how they think they are coming across to others (see Leigh and Clark [12] and Norton and Abbott [23] for reviews). As a result, there may be a reduction in outward directed attention [12, 23], which is likely to affect concentration on the teacher and classroom activities. Indirect support for this suggestion comes from the finding that self-reported concentration in class improved on average from 43% to 85% (with 0% indicating ‘*totally unable to concentrate*’) at follow-up in a case series of Cognitive Therapy for Adolescent Social Anxiety Disorder (CT-SAD-A) [24], implying that social anxiety and concentration may be inversely related to each other.

### Concentration and educational achievement

The ability to concentrate is important for learning and for academic success. Focused attention is needed in order to listen to information from the teacher, to complete classroom assignments, and to engage in group learning activities. Empirical evidence supports this assertion. For example, in a US sample of young people aged 6 to 13 years, the correlations between measures of basic cognitive skills such as attention and school achievement were large and of comparable magnitude to those between measures of fluid intelligence and school achievement [3]. Similarly, in a sample of 231 German adolescents (*M* age: 16.82 years), sustained attention was found to be a significant predictor of school grades over and above intelligence [11]. Furthermore, studies consistently indicate that young people with attention problems report poorer academic outcomes than their healthy peers [25–28]. Taken together, findings suggest that concentration has a direct association with educational achievement.

### Study aims

The aim of the present study was to test whether adolescent social anxiety is linked to poor educational achievement through a reduced ability to concentrate in class. It was hypothesized that baseline social anxiety symptoms would predict poorer concurrent concentration, which in turn, would predict poorer objective educational achievement 4–6 months after baseline assessment (Hypothesis 1). Given findings have been mixed regarding the effect of social anxiety symptoms on academic achievement, we also included a direct pathway in the model to be tested. It was hypothesized that social anxiety symptoms would predict poorer educational achievement at 4–6-month follow-up (Hypothesis 2). The objective assessment of educational achievement overcomes issues of common method variance, whereby associations are inflated because all measures are self-reported. Baseline educational achievement was included to rule out the possibility that observed prospective associations could be explained by concurrent correlations of relatively stable levels of social anxiety and academic achievement. Depression symptoms were included as a covariate in the analysis to rule out the possibility that any observed associations between social anxiety symptoms, concentration, and educational achievement are an artefact of depressive symptoms. Whilst not a focus of the present study, gender differences in key variables and the associations amongst them were examined, because some studies have indicated a gender difference in the effect of social anxiety on academic functioning, with a greater impact in girls than boys; for example, in a group of adolescents with a diagnosis of SAD girls reported having greater difficulty coping with their studies than boys [29].

## Methods

### Participants and procedures

Participants aged 11–14 years were recruited from two state-funded secondary schools in London. Participants were excluded if they were aged 15 or above (*n* = 1) or had difficulties reading or speaking English (*n* = 0). Ethics approval was obtained from the Central University Research Ethics Committee at the University of Oxford (Reference number: R54283/RE001). There were no other exclusion criteria. Parent opt-out consent and child written assent were obtained prior to data collection.

Before data collection, the research team explained the study to Years 7–9 students during tutor time. Students and their parents received an information sheet, an opt-out form, a prepaid envelope, and contact details of the research team. Information packs were sent to parents via the school intranet. Seven-hundred and eighteen students (360 from School 1 and 358 from School 2) were invited to take part in this study.

Of the 718 students, 617 (87%; 304 from School 1 and 313 from School 2) participated in the data collection. Data collection was conducted at least two weeks after the distribution of information packs. They completed questionnaires assessing social anxiety symptoms, depressive symptoms, and classroom concentration. In School 1, baseline educational achievement data was recorded in December 2017, self-report questionnaires were completed in February 2018, and outcome educational achievement data was recorded in December 2018. In School 2, baseline educational achievement data was recorded in September 2017, self-report questionnaires were completed in December 2017, and outcome educational achievement data was recorded in July 2018.

The final sample consists of 509 participants, because educational achievement data of Year 9 students in School 1 was not available (*n* = 107) and one participant from School 2 was excluded because they were aged 15 or above (*n* = 1). The sample from School 1 consisted of 197 young people (50% girls; *M* age = 12.54 years (*SD* = 0.58). 17% of the pupils were eligible for free school meals. 91% of them reported English as their first language. These figures are slightly higher than national statistics (UK Statistics Authority 2018). 3% of the students received special educational needs support, which is 8% below the national average. The sample from School 2 comprised of 312 adolescents (53% girls; *M* age = 12.91 (*SD* = 0.89). 10% of the students were eligible for free school meals, which is 4% below the national average (UK Statistics Authority 2018). Consistent with the national average, 82% of them reported English as their first language. Percentage of pupils who received special educational needs support (20%) was higher than the national average (10%). Pupils from School 2 were older (*p* < .001) and reported more social anxiety symptoms (*p* < .01) than those from School 1 (See S2 Table).

### Measures

#### Social anxiety symptoms

The Liebowitz Social Anxiety Scale for Children and Adolescents, self-report version (LSAS-CA-SR) is a measure of social anxiety symptoms in young people aged 7–18 years [30]. This scale assesses fear and avoidance in 24 social and performance situations, with a 0–3 rating (‘*none*’, ‘*mild*’, ‘*moderate*’ and ‘*severe*’). The total score is obtained by adding up the 48 items (range: 0–144). It demonstrates acceptable reliability (e.g. internal consistency reported between .95 –.97) and validity [22]. The self-report version of the scale was used in the present study [31]. The internal consistency of this scale in this sample was α = .96.

#### Depression symptoms

The Short Mood and Feelings Questionnaire (SMFQ) is a 13-item self-report questionnaire assessing different aspects of depressive symptoms in young people aged 6–17 years [32]. Each item on the scale ranges from 0 (*not true*) to 2 (*true*). The total score is derived by summing the ratings for each item. Psychometric properties has been shown to be acceptable (e.g. internal consistency: .88 –.89) [33]. The internal consistency of this scale in this sample was α = .91.

#### Concentration in class

Concentration in class was assessed with a single item self-report scale [24] on which the young person indicates, on a 0–100 scale, how well they have been able to concentrate on what the teacher is saying and what they have been learning in class in the preceding week (with 0 indicating ‘*not at all able to concentrate*’ and 100 ‘*totally able to concentrate*’).

#### Educational achievement

School 1 and 2 used slightly different metrics for quantifying participants’ educational achievement. School 1 provided us with students’ teacher-assessed average grade across subjects in December 2017 and their teacher-assessed average grade across subjects in December 2018. School 2 provided us with a single ‘difference score’, quantifying the difference between students’ teacher-predicted grades across subjects in September 2017 and their teacher-assessed average grade across subjects in July 2018. In order to examine the data from the two schools together, a difference score was calculated to measure educational achievement in School 1 as well as School 2, equal to: average grade in 2018 –average grade in 2017. Difference scores for each school were standardized before then aggregating into one ‘educational achievement’ variable.

### Data analysis

Analyses were undertaken in R [34]. All variables were checked for missingness. At item level, mean imputation was used where less than 5% of items were missing for a questionnaire. At variable level, data were missing for 12 participants on the LSAS-CA-SR, 57 participants on the SMFQ, 50 participants on the concentration measure, and 19 for educational achievement. To examine whether baseline variables (gender, LSAS-CA-SR, SMFQ, or concentration) were associated with missingness of the educational achievement variable, logistic regressions were conducted with these variables entered as predictors of educational achievement variable missingness. None emerged as a significant predictor. Furthermore, Little’s MCAR test was not significant (*p* > .05), indicating data was missing completely at random. For the mediation analysis, missing data at variable level was managed with a model estimation method (see below), for other analyses pairwise deletion was used.

Means and standard deviations of the main variables and correlations amongst them were examined for the group as a whole and separately for girls and boys. Fisher’s *r*-to-*z* tests were used to determine whether the magnitude of the association between variables differed between genders. Path analysis was undertaken with the ‘lavaan’ [35] package in R, to examine the study’s central research question of whether the association between social anxiety and educational achievement is mediated by concentration in class. In the language of path analysis, mediation corresponds to an indirect effect of an independent variable (social anxiety symptom score) on a dependent variable (educational achievement) that passes through one or more mediator variables (concentration measure) [36, 37]. The indirect effect is calculated by multiplying the paths that constitute the effect. Missing data were handled using full information maximum likelihood (FIML) estimation, which adjusts the likelihood function so that each case contributes information on the variables that are observed. Bootstrapping with 1000 replications was applied to derive more accurate standard errors and bias-corrected confidence intervals were computed. All variables were standardized prior to analysis.

## Results

### Descriptive statistics

Descriptive statistics (means, standard deviations, and correlations) of the whole sample for the main variables are presented in Table 1.

**Table 1:** Descriptive statistics and Pearson’s correlations of main study variables ‡.

	Depression symptoms	Concentration	Educational achievement	Mean (SD)
Social anxiety symptoms	0.60***	-0.39***	0.05	40.11 (28.37)
Depression symptoms		-0.47***	-0.01	6.83 (6.33)
Concentration			0.18***	68.22 (24.83)

Notes

^‡^ Unstandardized means and standard deviations are presented for social anxiety symptoms, concentration, and depression symptoms. Mean and standard deviation are not provided for Attainment because these scores were standardized to combine the difference scores for the two schools

* *p* < .05; ** *p* < .01

***: *p* < .001.

Descriptive statistics for the sample split by gender are shown in Table 2. As can be seen, girls endorsed more social anxiety and depression symptoms compared to boys, consistent with epidemiological data [38]. Self-reported concentration did not differ by gender, however girls showed greater improvements in educational achievement over time compared to boys (mean of standardized attainment metric for girls compared to boys: 0.13 vs. -0.15). Despite the group differences, the pattern of associations amongst the main variables was similar between genders. Fisher’s *r*-to-*z* transformation to compare correlations between genders indicated no significant differences between any pair of correlations (*p* > .05 for all comparisons). Due to the gender differences observed on three of the four main variables, but the patterns of association not differing, gender was included as a covariate in the path analysis.

**Table 2:** Descriptive statistics and Pearson’s correlations of main study variables split by gender (correlation coefficients for females [N = 274] presented above the diagonal, for males [N = 235] below) ‡.

	Social anxiety symptoms	Depression symptoms	Concentration	Educational achievement	Females Mean (SD)	Males Mean (SD)	*t*-test
Social anxiety symptoms	1	0.60***	-0.44***	-0.03	45.01 (30.81)	34.32 (23.99)	*t*(495) = 4.26 ***
Depression symptoms	0.56***	1	-0.48***	-0.08	8.39 (7.01)	5.08 (4.93)	*t*(450) = 5.76 ***
Concentration	-0.29***	-0.47***	1	0.23***	66.47 (25.49)	70.18 (23.97)	*t*(457) = -1.60
Educational achievement	0.10	-0.01	0.15*	1	0.13 (1.00)	-0.15 (0.98)	*t*(488) = 3.10 **

Notes

^‡^ Unstandardized means and standard deviations are presented for social anxiety symptoms, concentration, and depression symptoms, standardized means and standard deviations are provided for Attainment

* *p* < .05

**: *p* < .01

***: *p* < .001.

### Mediation model

**Fig 1:**
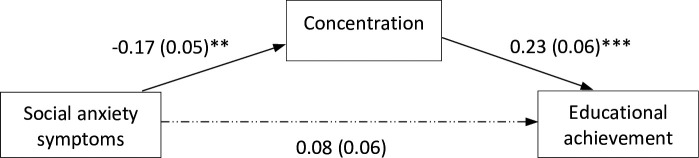
Mediation model testing the direct effect of social anxiety on educational achievement and the indirect effect via concentration (depression symptoms and gender were included as covariates for the dependent variable but are not shown here for ease of interpretation). Values presented are standardized path coefficients, values presented in parentheses are standard errors. ** *p* < .01; *** *p* < .001.

We tested a mediation model (see Fig 1) in which baseline social anxiety symptoms were examined as a predictor of educational achievement. Concentration was included as a mediator of the association. Baseline depression symptoms and gender were included as covariates.

As can be seen in Fig 1, baseline social anxiety symptoms did not predict educational achievement directly (*b* = 0.08 [95% CI: -0.04, 0.21], *SE* = 0.06, *p* > .05) nor was the total effect significant (*b* = 0.04 [95% CI: -0.18, 0.17], *SE* = 0.06, *p* > .05). However, social anxiety symptoms were associated with concentration in the classroom (*b* = -0.17 [95% CI: -0.29, -0.07], *SE* = 0.05, *p* < .01), with greater social anxiety associated with poorer concentration. Furthermore, concentration was associated with educational achievement (*b* = 0.23 [95% CI: 0.11, 0.35], *SE* = 0.06, *p* < .001), by which better concentration was linked to better achievement. As such, there was a significant indirect effect of social anxiety symptoms on educational achievement one year later, through concentration in the classroom (*b* = -0.04 [95% CI: -0.08, -0.02], *SE* = 0.02, *p* < .01). Higher baseline social anxiety symptoms were linked to poorer concentration in class which in turn was associated with lower educational achievement 9–12 months later.

The covariate depression symptoms was not found to have a significant direct association with educational achievement (*b* = 0.02, *SE* = 0.07 [95% CI: -0.11, 0.14], *p* > .05), but it was significantly associated with concentration (*b* = -0.38 [95% CI: -0.49, -0.26], *SE* = 0.06, *p* < .001). Gender showed a direct association with educational achievement (*b* = -0.34 [95% CI: -0.53, -0.12], *SE* = 0.10, *p* < .01), with girls showing better educational achievement than boys, but gender was not associated with concentration (*b* = -0.09, *SE* = 0.09 [95% CI: -0.26, 0.08], *p* > .05).

As a follow-up analysis to examine the possibility of school-level differences in the findings, the mediation model was rerun with school included as a covariate, in addition to depression symptoms and gender. The findings were unchanged, and school was not associated with concentration (*b* = -0.06, *SE* = 0.09 [95% CI: -0.24, 0.10], *p* > .05) or educational achievement (*b* = 0.10, *SE* = 0.11 [95% CI: -0.10, 0.31], *p* > .05).

## Discussion

Educational achievement in the adolescent years is a key predictor of a range of life outcomes [2]. However, adolescence is also the time when social anxiety typically first occurs, and the present study aimed to examine the hypothesis that social anxiety symptoms interfere with learning abilities leading to reduced educational achievement. Although social anxiety symptoms was not found to have a direct effect on later educational achievement, in line with the hypothesis, an indirect association was observed via reduced concentration in the classroom.

The finding that social anxiety symptoms are positively associated with concentration is consistent with existing theories on the role of self-focused attention in social anxiety [39–41]. It is possible that when adolescents feel socially anxious, their attention shifts towards internal information, such as negative thoughts (e.g. “*I will blush when I have to answer a question*”) and anxious feelings (e.g. hot cheeks) [42], with less attention directed towards classroom activities [23]. This in turn may limit adolescents’ ability to make the most of these learning opportunities, which then affects their educational achievement. The suggestion is consistent with the proposal of Derakshan and Eysenck [43] that anxiety has adverse effects on cognitive performance due to increased attention given over to task-irrelevant stimuli (such as negative cognitions and anxious feelings).

Current findings indicate some specificity in the indirect association between social anxiety symptoms and educational achievement. Although depression has been shown to be associated with social anxiety [44], attention difficulties [45], and educational achievement [10], it was observed that the indirect association between social anxiety symptoms and educational achievement via concentration was significant when controlling for baseline depression symptoms. This speaks against the idea that depression symptoms may account for the association, and instead supports the proposal that social anxiety is a particular vulnerability factor for poorer school achievement in adolescents. Future studies could examine whether the effect is linked to anxiety in general, or specific to social anxiety.

We did not find evidence of a direct or total effect of social anxiety symptoms on educational achievement, which tallies with the findings of Scanlon and colleagues [18] in their similar study. Whilst it is now widely accepted that the lack of a direct or total effect is not inconsistent with the presence of an indirect effect [36, 37, 46, 47], it would be interesting to consider the factors underlying the observation, such as subgroup effects, in future studies.

Possible gender differences across key study variables were examined. The gender differences observed in three of the four variables were in line with previous research in terms of: higher emotional symptoms in females compared to males [48] and better educational achievement on average in girls than boys [49]. Furthermore, the absence of a gender difference in levels of concentration is also consistent with empirical findings (e.g. [50]). However, differences were not found in the pattern of associations amongst these variables, suggesting that the relationships operate in a similar manner amongst girls and boys.

The current study has strengths. Methodological strengths include the use of a prospective study design, statistical control for baseline educational achievement and depressive symptoms in the mediation model, and the use of objective educational achievement measures. However, several limitations in this study should be considered. The main limitation is the lack of control for additional factors that may explain the variances of educational achievement, such as peer support, parental educational level, and previous diagnoses of anxiety and/or depressive disorders [4–6]. The use of a single-item measure for classroom concentration is prone to random measurement error and may underestimate associations, and it will be important that future studies address this limitation. Furthermore, the measures of social anxiety symptoms and concentration were administered at the same time point and so we cannot draw conclusions about the temporal precedence of these variables. A further limitation is that the assessments were conducted at different points of the academic year in two different schools. Theoretically it is possible that the strength of association between variables may vary over the academic year due to external factors, such as academic demand and the time lag between symptoms measured and examinations. However, the similar pattern of associations that was observed in the two schools speaks against this concern.

The current findings point to a number of avenues for future research. Taking our findings together with those from the cross-sectional study of Scanlon, Del Toro [18], who observed an indirect association between social anxiety symptoms and science achievement via reduced social engagement, it seems likely that multiple indirect pathways may operate between social anxiety and educational achievement. Future studies measuring more than one putative mediator will be valuable to tease apart the interrelationships amongst these variables. Related to this, it may be that factors such as concentration and social engagement are not only affected by social anxiety, but also contribute to increased social anxiety in the classroom, creating a vicious cycle. For example, difficulty concentrating and learning in class may exacerbate fears about getting an answer wrong and making a fool of oneself. Examining this in studies with multiple waves of measurement could be informative. In addition, concentration and attentional processes are maturing during adolescence [51, 52] and social anxiety typically shows age-related increases during this time [53] and therefore it is possible that the associations amongst social anxiety symptoms, concentration, and educational achievement may vary across this developmental period. Furthermore, the determinants of educational achievement may vary across childhood and adolescence. It may be that the relative contribution of social anxiety is greater in adolescence than earlier in life, whilst systemic factors such as parenting style [54, 55] may play a greater role earlier in childhood. Future studies with larger sample sizes and a wider age range could examine these possibilities.

The study findings have implications for education and clinical practices. In schools, an increased awareness of the impact of social anxiety on learning and concentration should be encouraged amongst teachers. With regards to clinical practice, current results suggest psychological interventions for adolescent social anxiety may have the potential to improve educational outcomes of affected individuals. There is evidence that adolescents experience reductions in social anxiety and improved concentration after receiving a course of CT-SAD-A [24]. These changes may have a beneficial effect on academic achievement. Further research is needed to examine whether CT-SAD-A leads to an improvement of educational achievement in adolescents.

## Conclusions

Academic success paves the way for success in many areas of life [2], and it is the adolescent years when critical examinations are undertaken. Understanding risk factors for reduced educational achievement is therefore an important endeavour. The present study demonstrated that social anxiety symptoms reduce adolescents’ ability to concentrate in class which, in turn, impacts on their educational achievement over time, over and above baseline educational achievement and depression symptoms. Findings point to the fundamentally social nature of school learning environments, the challenges socially anxious adolescents will face trying to learn in school, and the need for education providers and clinicians to consider the effect of social anxiety symptoms on concentration and learning.

## Supporting information

S1 Table Results of independent sample t-tests between females’ and males’ scores. (DOC)

S2 Table Results of independent sample t-tests between school 1 and school 2 scores. (DOC)

S3 Table Measures of different constructs in the survey. (DOC)

## References

[1] Spinks R, Arndt S, Caspers K, Yucuis R, McKirgan LW, Pfalzgraf C, et al. School achievement strongly predicts midlife IQ. Intelligence. 2007;35(6):563–7.

[2] Cutler DM, Lleras-Muney A. Education and health: insights from international comparisons. National Bureau of Economic Research; 2012. Report No.: 0898–2937.

[3] Luo D, Thompson LA, Detterman DK. The criterion validity of tasks of basic cognitive processes. Intelligence. 2006;34(1):79–120.

[4] Poropat AE. A meta-analysis of the five-factor model of personality and academic performance. Psychological Bulletin. 2009;135(2):322–38. 10.1037/a0014996 19254083

[5] Dotterer AM, Lowe K. Classroom context, school engagement, and academic achievement in early adolescence. Journal of Youth and Adolescence. 2011;40(12):1649–60. 10.1007/s10964-011-9647-5 21400208

[6] Steinmayr R, Heyder A, Naumburg C, Michels J, Wirthwein L. School-related and individual predictors of subjective well-being and academic achievement. Frontiers in Psychology. 2018;9(2631). 10.3389/fpsyg.2018.02631 30622497 PMC6308923

[7] de Lijster JM, Dieleman GC, Utens EMWJ, Dierckx, Wierenga M Verhulst FC, et al. Social and academic functioning in adolescents with anxiety disorders: A systematic review. Journal of affective disorders. 2018;230:108–17. 10.1016/j.jad.2018.01.008 29407534

[8] Kessler RC, Berglund P, Demler O, Jin R, Merikangas KR, Walters EE. Lifetime prevalence and age-of-onset distributions of DSM-IV disorders in the National Comorbidity Survey Replication. Arch Gen Psychiatry. 2005;62(6):593–602. 10.1001/archpsyc.62.6.593 15939837

[9] Vilaplana-Pérez A, Pérez-Vigil A, Sidorchuk A, Brander G, Isomura K, Hesselmark E, et al. Much more than just shyness: the impact of social anxiety disorder on educational performance across the lifespan. Psychological Medicine. 2020:1–9. 10.1017/S0033291719003908 31907098 PMC8108394

[10] Riglin L, Petrides KV, Frederickson N, Rice F. The relationship between emotional problems and subsequent school attainment: A meta-analysis. Journal of Adolescence. 2014;37(4):335–46. 10.1016/j.adolescence.2014.02.010 24793380

[11] Steinmayr R, Ziegler M, Träuble B. Do intelligence and sustained attention interact in predicting academic achievement? Learning and Individual Differences. 2010;20(1):14–8.

[12] Leigh E, Clark DM. Understanding Social Anxiety Disorder in Adolescents and Improving Treatment Outcomes: Applying the Cognitive Model of Clark and Wells (1995). Clinical Child and Family Psychology Review. 2018. 10.1007/s10567-018-0258-5 PMC6447508 29654442

[13] Ranta K, Kaltiala-Heino R, Rantanen P, Marttunen M. Social phobia in Finnish general adolescent population: Prevalence, comorbidity, individual and family correlates, and service use. Depression and Anxiety. 2009;26(6):528–36. 10.1002/da.20422 19170089

[14] Soohinda G, Sampath H. Social phobia among school students—prevalence, demographic correlates and socio-academic impairment. Journal of Indian Association for Child & Adolescent Mental Health. 2016;12(3).

[15] Nail JE, Christofferson J, Ginsburg GS, Drake K, Kendall PC, McCracken JT, et al. Academic impairment and impact of treatments among youth with anxiety disorders. Child & Youth Care Forum. 2015;44(3):327–42.

[16] Strahan EY. The effects of social anxiety and social skills on academic performance. Personality and Individual Differences. 2003;34(2):347–66.

[17] Ranta K, La Greca AM, Kaltiala-Heino R, Marttunen M. Social phobia and educational and interpersonal impairments in adolescence: A prospective study. Child Psychiatry and Human Development. 2016;47(4):665–77. 10.1007/s10578-015-0600-9 26514560

[18] Scanlon CL, Del Toro J, Wang M-T. Socially anxious science achievers: the roles of peer social support and social engagement in the relation between adolescents’ social anxiety and science achievement. Journal of Youth and Adolescence. 2020;49(5):1005–16. 10.1007/s10964-020-01224-y 32206958

[19] Moran A. Concentration: Attention and performance. In: Murphy SM, editor. The Oxford Handbook of Sport and Performance Psychology. Oxford, UK: Oxford University Press; 2012. p. 117–30.

[20] Eysenck MW, Derakshan N, Santos R, Calvo MG. Anxiety and cognitive performance: attentional control theory. Emotion. 2007;7(2):336–53. 10.1037/1528-3542.7.2.336 17516812

[21] Robinson OJ, Vytal K, Cornwell BR, Grillon C. The impact of anxiety upon cognition: perspectives from human threat of shock studies. Frontiers in Human Neuroscience. 2013;7:203–. 10.3389/fnhum.2013.00203 23730279 PMC3656338

[22] Masia-Warner C, Storch EA, Pincus DB, Klein RG, Heimberg RG, Liebowitz MR. The Liebowitz Social Anxiety Scale for Children and Adolescents: An Initial Psychometric Investigation. Journal of the American Academy of Child & Adolescent Psychiatry. 2003;42(9):1076–84. 12960707 10.1097/01.CHI.0000070249.24125.89

[23] Norton AR, Abbott MJ. Self-focused cognition in social anxiety: A review of the theoretical and empirical literature. Behaviour Change. 2016;33(1):44–64.

[24] Leigh E, Clark DM. Cognitive Therapy for Social Anxiety Disorder in Adolescents: A Development Case Series. Behavioural and Cognitive Psychotherapy. 2016;44(1):1–17. 10.1017/S1352465815000715 26640031 PMC5964462

[25] Polderman T, Boomsma D, Bartels M, Verhulst F, Huizink A. A systematic review of prospective studies on attention problems and academic achievement. Acta psychiatrica Scandinavica. 2010;122(4):271–84. 10.1111/j.1600-0447.2010.01568.x 20491715

[26] Rabiner DL, Godwin J, Dodge KA. Predicting academic achievement and attainment: The contribution of early academic skills, attention difficulties, and social competence. School Psychology Review. 2016;45(2):250–67.

[27] Sayal K, Washbrook E, Propper C. Childhood behavior problems and academic outcomes in adolescence: longitudinal population-based study. Journal of the American Academy of Child & Adolescent Psychiatry. 2015;54(5):360–8. e2. 10.1016/j.jaac.2015.02.007 25901772

[28] Valiente C, Eisenberg N, Spinrad TL, Haugen R, Thompson MS, Kupfer A. Effortful control and impulsivity as concurrent and longitudinal predictors of academic achievement. The Journal of Early Adolescence. 2013;33(7):946–72.

[29] Mehtalia K, Vankar GK. Social anxiety in adolescents. Indian Journal of Psychiatry. 2004;46(3):221–7. 21224903 PMC2951647

[30] Masia-Warner C, Klein RG, Liebowitz MR. The Liebowitz Social Anxiety Scale for Children and Adolescents (LSAS-CA). 1999. 10.1097/01.CHI.0000070249.24125.89 12960707

[31] Schmits E, Heeren A, Quertemont E. The self-report Version of the LSAS-CA: Psychometric Properties of the French Version in a non-clinical adolescent sample. Psychologica Belgica. 2014;54(2):181–98.

[32] Angold A, Costello EJ, Messer SC, Pickles A, Winder F, Silver D. The development of a short questionnaire for use in epidemiological studies of depression in children and adolescents. International Journal of Methods in Psychiatric Research. 1995;5:237–49.

[33] Thabrew H, Stasiak K, Bavin LM, Frampton C, Merry S. Validation of the Mood and Feelings Questionnaire (MFQ) and Short Mood and Feelings Questionnaire (SMFQ) in New Zealand help-seeking adolescents. International Journal of Methods in Psychiatric Research. 2018;27(3):e1610. 10.1002/mpr.1610 29465165 PMC6877137

[34] R Core Team. R: A language and environment for statistical computing. R Foundation for Statistical Computing. Vienna. Austria 2019.

[35] Rosseel Y. lavaan: An R Package for Structural Equation Modeling. 2012. 2012;48(2):36.

[36] Agler R, De Boeck P. On the Interpretation and Use of Mediation: Multiple Perspectives on Mediation Analysis. Frontiers in Psychology. 2017;8(1984). 10.3389/fpsyg.2017.01984 29187828 PMC5694788

[37] MacKinnon DP, Fairchild AJ, Fritz MS. Mediation Analysis. Annual Review of Psychology. 2007;58(1):593–614. 10.1146/annurev.psych.58.110405.085542 16968208 PMC2819368

[38] Asher M, Aderka IM. Gender differences in social anxiety disorder. Journal of Clinical Psychology. 2018;74(10):1730–41. 10.1002/jclp.22624 29667715

[39] Clark DM, Wells A. A cognitive model of social phobia. In: Heimberg G, M. R. Liebowitz MR, Hope D, Scheier F, editors. Social phobia: Diagnosis, assessment, and treatment. New York: The Guilford Press; 1995. p. 69–93.

[40] Hofmann SG. Cognitive factors that maintain social anxiety disorder: A comprehensive model and its treatment implications. Cognitive behaviour therapy. 2007;36(4):193–209. 10.1080/16506070701421313 18049945 PMC2151931

[41] Rapee RM, Heimberg RG. A cognitive-behavioral model of anxiety in social phobia. Behaviour Research & Therapy. 1997;35(8):741–56. 10.1016/s0005-7967(97)00022-3 9256517

[42] Kley H, Tuschen-Caffier B, Heinrichs N. Safety behaviors, self-focused attention and negative thinking in children with social anxiety disorder, socially anxious and non-anxious children. Journal of Behavior Therapy and Experimental Psychiatry. 2012;43(1):548–55. 10.1016/j.jbtep.2011.07.008 21831344

[43] Derakshan N, Eysenck MW. Anxiety, processing efficiency, and cognitive performance: New developments from attentional control theory. European Psychologist. 2009;14(2):168–76.

[44] Cummings CM, Caporino NE, Kendall PC. Comorbidity of anxiety and depression in children and adolescents: 20 years after. Psychol Bull. 2014;140(3):816–45. 10.1037/a0034733 24219155 PMC4006306

[45] Vilgis V, Silk TJ, Vance A. Executive function and attention in children and adolescents with depressive disorders: a systematic review. European Child & Adolescent Psychiatry. 2015;24(4):365–84. 10.1007/s00787-015-0675-7 25633323

[46] Zhao X, Lynch J, xa G, Chen Q, John Deightonserved as e, et al. Reconsidering Baron and Kenny: Myths and Truths about Mediation Analysis. Journal of Consumer Research. 2010;37(2):197–206.

[47] Hayes AF, Preacher KJ, Myers TA. Mediation and the estimation of indirect effects in political communication research. Sourcebook for political communication research: Methods, measures, and analytical techniques. 2011;23(1):434–65.

[48] Faravelli C, Alessandra Scarpato M, Castellini G, Lo Sauro C. Gender differences in depression and anxiety: The role of age. Psychiatry research. 2013;210(3):1301–3. 10.1016/j.psychres.2013.09.027 24135551

[49] Gorard S, Rees G, Salisbury J. Investigating the Patterns of Differential Attainment of Boys and Girls at School. British Educational Research Journal. 2001;27(2):125–39.

[50] Solianik R, Brazaitis M, Skurvydas A. Sex-related differences in attention and memory. Medicina. 2016;52(6):372–7. 10.1016/j.medici.2016.11.007 27932195

[51] McAvinue LP, Habekost T, Johnson KA, Kyllingsbæk S, Vangkilde S, Bundesen C, et al. Sustained attention, attentional selectivity, and attentional capacity across the lifespan. Attention, Perception, & Psychophysics. 2012;74(8):1570–82. 10.3758/s13414-012-0352-6 22825931

[52] Boelema SR, Harakeh Z, Ormel J, Hartman CA, Vollebergh WA, van Zandvoort MJ. Executive functioning shows differential maturation from early to late adolescence: Longitudinal findings from a TRAILS study. Neuropsychology. 2014;28(2):177. 10.1037/neu0000049 24364395

[53] Westenberg PM, Bokhorst CL, Miers AC, Sumter SR, Kallen VL, van Pelt J, et al. A prepared speech in front of a pre-recorded audience: subjective, physiological, and neuroendocrine responses to the Leiden Public Speaking Task. Biological Psychology. 2009;82:116–24. 10.1016/j.biopsycho.2009.06.005 19576261

[54] Pettit GS, Yu T, Dodge KA, Bates JE. A developmental process analysis of cross-generational continuity in educational attainment. Merrill Palmer Q (Wayne State Univ Press). 2009;55(3):250–84. 10.1353/mpq.0.0022 22822286 PMC3400156

[55] Cheesman R, Hunjan A, Coleman JRI, Ahmadzadeh Y, Plomin R, McAdams TA, et al. Comparison of adopted and nonadopted individuals reveals gene–environment interplay for education in the UK Biobank. Psychological Science. 2020;31(5):582–91. 10.1177/0956797620904450 32302253 PMC7238511

