# Supplementary material for: Is concentration an indirect link between social anxiety and educational achievement in adolescents?
Source: PLoS One. 2021 May 14;16(5):e0249952. doi: 10.1371/journal.pone.0249952 (PMC8121284; doi:10.1371/journal.pone.0249952)
Supplement: S1 Table — (DOC) [file pone.0249952.s001.doc]

| S1 Table: Results of independent sample t-tests between females’ and males’ scores | | | |
| --- | --- | --- | --- |
| Variable | Mean (SD)  Females | Mean (SD)  Males | *t*-tests and *p*-values |
| 1. Age1 | 12.81 (0.83) | 12.72 (0.77) | *t*(494) = 1.22, *p* = .22 |
| 2. LSAS1 | 45.01 (30.80) | 34.32 (24.00) | *t*(495) = 4.26 *** |
| 3. SMFQ1 | 8.40 (7.01) | 5.08 (4.93) | *t*(450) = 5.76 *** |
| 4. CON1 | 66.47 (25.50) | 70.18 (24.00) | *t*(457) = -1.60, *p* = .11 |
| 5. ACH1 | 65.97 (13.00) | 64.66 (11.40) | *t*(176) = 0.72, *p* = .47 |
| 6. ACH2 | 66.06 (13.50) | 64.68 (11.20) | *t*(176) = 0.74, *p* = .46 |
| 7. ATT | 0.07 (0.84) | -0.31 (0.87) | *t*(310) = 3.88 *** |

*Note.* *** indicates *p* < .05. ** indicates *p* < .01. *** indicates *p* < .001. *M* = mean, *SD* = standard deviation. Age1 = Age (baseline), LSAS1 = LSAS total score (baseline), SMFQ1 = SMFQ total score (baseline), CON1 = concentration (baseline), ACH1 = academic achievement (baseline), ACH2 = academic achievement (follow-up). ATT = academic attainment.
