# Supplementary material for: Is concentration an indirect link between social anxiety and educational achievement in adolescents?
Source: PLoS One. 2021 May 14;16(5):e0249952. doi: 10.1371/journal.pone.0249952 (PMC8121284; doi:10.1371/journal.pone.0249952)
Supplement: S2 Table — (DOC) [file pone.0249952.s002.doc]

| S2 Table: Results of independent sample t-tests between school 1 and school 2 scores | | | |
| --- | --- | --- | --- |
| Variable | Mean (SD)  School 1 | Mean (SD)  School 2 | *t*-tests and *p*-values |
| 1. Age1 | 12.54 (0.58) | 12.91 (0.89) | *t*(494) = -5.24 *** |
| 2. LSAS1 | 35.17 (27.10) | 43.19 (28.80) | *t*(495) = -3.09 ** |
| 3. SMFQ1 | 6.14 (6.36) | 7.24 (6.29) | *t*(450) = -1.80, *p* = .07 |
| 4. CON1 | 70.65 (25.70) | 66.68 (24.20) | *t*(457) = 1.67, *p* = .09 |

*Note.* *** indicates *p* < .05. ** indicates *p* < .01. *** indicates *p* < .001. *M* = mean, *SD* = standard deviation. Age1 = Age (baseline), LSAS1 = LSAS total score (baseline), SMFQ1 = SMFQ total score (baseline), CON1 = concentration (baseline).
