# Supplementary material for: Is concentration an indirect link between social anxiety and educational achievement in adolescents?
Source: PLoS One. 2021 May 14;16(5):e0249952. doi: 10.1371/journal.pone.0249952 (PMC8121284; doi:10.1371/journal.pone.0249952)
Supplement: S3 Table — (DOC) [file pone.0249952.s003.doc]

| S3 Table: Measures of different constructs in the survey | |
| --- | --- |
| Construct | Measure |
| Social anxiety symptoms | LSAS-CA-SR |
| Depression symptoms | SMFQ |
| Concentration | Self-report item |
| Age and gender | Self-report items |

*Note.* LSAS-CA-SR: Liebowitz Social Anxiety Scale for Children and Adolescent-Self-report. SMFQ: Short Mood and Feeling Questionnaire.
